# Supplementary material for: End Sequence Analysis Toolkit (ESAT) expands the extractable information from single-cell RNA-seq data
Source: Genome Res. 2016 Oct;26(10):1397–410. doi: 10.1101/gr.207902.116 (PMC5052061; doi:10.1101/gr.207902.116)
Supplement: Supplemental Material [file supp_gr.207902.116_Supp_Materials.tar.gz › ESAT-esat_v0.1/doc/html/classbroad_1_1pda_1_1seq_1_1fastq_1_1_fastq_parser.html]

ESAT: broad.pda.seq.fastq.FastqParser Class Reference


ESAT


- Main Page
- Related Pages
- Packages
- Classes
- Files

- Class List
- Class Index
- Class Hierarchy
- Class Members

All Classes Namespaces Files Functions Variables Enumerator Pages

- broad
- pda
- seq
- fastq
- FastqParser

Public Member Functions |
List of all members

broad.pda.seq.fastq.FastqParser Class Reference

Inheritance diagram for broad.pda.seq.fastq.FastqParser:

[legend]

Collaboration diagram for broad.pda.seq.fastq.FastqParser:

[legend]

|  |  |
| --- | --- |
| Public Member Functions | |
|  | FastqParser (File fastqFile) throws IOException |
|  | |
|  | FastqParser () |
|  | |
| void | start (File fastqParser) throws IOException |
|  | |
| void | start (BufferedReader br) throws IOException |
|  | |
| void | convertToFasta (String save) throws IOException |
|  | |
| void | convertToNumberedFasta (String save) throws IOException |
|  | |
| Collection< FastqSequence > | parse (File file) throws IOException |
|  | |
| Collection< FastqSequence > | getSequences () throws IOException |
|  | |
| File[] | writeChunks (String save, int chunkSize) throws IOException |
|  | |
| int | getNumberOfSequences () |
|  | |
| boolean | hasNext () |
|  | |
| FastqSequence | next () |
|  | |
| void | close () throws IOException |
|  | |
| void | remove () |
|  | |

## Constructor & Destructor Documentation

|  |  |  |  |  |  |
| --- | --- | --- | --- | --- | --- |
| broad.pda.seq.fastq.FastqParser.FastqParser | ( | File | *fastqFile* | ) | throws IOException |

**Deprecated:**
:   This constructure is highly discouraged as it opens a reader. Use the empty constructor instead and set the file to read, then call start.

    Parameters
    :   |  |  |
        | --- | --- |
        | fastqFile |  |

    Exceptions
    :   |  |  |
        | --- | --- |
        | IOException |  |

|  |  |  |  |  |
| --- | --- | --- | --- | --- |
| broad.pda.seq.fastq.FastqParser.FastqParser | ( |  | ) |  |

## Member Function Documentation

|  |  |  |  |  |
| --- | --- | --- | --- | --- |
| void broad.pda.seq.fastq.FastqParser.close | ( |  | ) | throws IOException |

|  |  |  |  |  |  |
| --- | --- | --- | --- | --- | --- |
| void broad.pda.seq.fastq.FastqParser.convertToFasta | ( | String | *save* | ) | throws IOException |

|  |  |  |  |  |  |
| --- | --- | --- | --- | --- | --- |
| void broad.pda.seq.fastq.FastqParser.convertToNumberedFasta | ( | String | *save* | ) | throws IOException |

|  |  |  |  |  |
| --- | --- | --- | --- | --- |
| int broad.pda.seq.fastq.FastqParser.getNumberOfSequences | ( |  | ) |  |

|  |  |  |  |  |
| --- | --- | --- | --- | --- |
| Collection<FastqSequence> broad.pda.seq.fastq.FastqParser.getSequences | ( |  | ) | throws IOException |

|  |  |  |  |  |
| --- | --- | --- | --- | --- |
| boolean broad.pda.seq.fastq.FastqParser.hasNext | ( |  | ) |  |

|  |  |  |  |  |
| --- | --- | --- | --- | --- |
| FastqSequence broad.pda.seq.fastq.FastqParser.next | ( |  | ) |  |

|  |  |  |  |  |  |
| --- | --- | --- | --- | --- | --- |
| Collection<FastqSequence> broad.pda.seq.fastq.FastqParser.parse | ( | File | *file* | ) | throws IOException |

|  |  |  |  |  |
| --- | --- | --- | --- | --- |
| void broad.pda.seq.fastq.FastqParser.remove | ( |  | ) |  |

|  |  |  |  |  |  |
| --- | --- | --- | --- | --- | --- |
| void broad.pda.seq.fastq.FastqParser.start | ( | File | *fastqParser* | ) | throws IOException |

|  |  |  |  |  |  |
| --- | --- | --- | --- | --- | --- |
| void broad.pda.seq.fastq.FastqParser.start | ( | BufferedReader | *br* | ) | throws IOException |

|  |  |  |  |
| --- | --- | --- | --- |
| File [] broad.pda.seq.fastq.FastqParser.writeChunks | ( | String | *save*, |
|  |  | int | *chunkSize* |
|  | ) |  | throws IOException |

---

The documentation for this class was generated from the following file:

- java/broad/pda/seq/fastq/FastqParser.java


---

Generated on Fri Aug 15 2014 15:11:02 for ESAT by  

 1.8.7
